# Inhibitory Control and Brain–Heart Interaction: An HRV-EEG Study

**DOI:** 10.3390/brainsci12060740

**Published:** 2022-06-05

**Authors:** Maria Daniela Cortese, Martina Vatrano, Paolo Tonin, Antonio Cerasa, Francesco Riganello

**Affiliations:** 1 Sant’Anna Institute, 88900 Crotone, Italy; d.cortese@isakr.it (M.D.C.); m.vatrano@isakr.it (M.V.); patonin18@gmail.com (P.T.); antonio.cerasa76@gmail.com (A.C.); 2 Institute for Biomedical Research and Innovation (IRIB), National Research Council of Italy (CNR), 98100 Messina, Italy

**Keywords:** inhibitory control, EEG, HRV, entropy, Central Autonomic Network

## Abstract

Background: Motor inhibition is a complex cognitive function regulated by specific brain regions and influenced by the activity of the Central Autonomic Network. We investigate the two-way Brain–Heart interaction during a Go/NoGo task. Spectral EEG ϑ, α powerbands, and HRV parameters (Complexity Index (CI), Low Frequency (LF) and High Frequency (HF) powers) were recorded. Methods: Fourteen healthy volunteers were enrolled. We used a modified version of the classical Go/NoGo task, based on Rule Shift Cards, characterized by a baseline and two different tasks of different complexity. The participants were divided into subjects with Good (GP) and Poor (PP) performances. Results: In the baseline, CI was negatively correlated with α/ϑ. In task 1, the CI was negatively correlated with the errors and α/ϑ, while the errors were positively correlated with α/ϑ. In task 2, CI was negatively correlated with the Reaction Time and positively with α, and the errors were negatively correlated with the Reaction Time and positively correlated with α/ϑ. The GP group showed, at baseline, a negative correlation between CI and α/ϑ. Conclusions: We provide a new combined Brain–Heart model underlying inhibitory control abilities. The results are consistent with the complementary role of α and ϑ oscillations in cognitive control.

## 1. Introduction

Inhibition has been identified as a common factor underlying performance in all-executive function tasks [1], with the prefrontal cortex acting as the principal source of inhibitory control in concert with the subcortical brain areas, putamen and subthalamic nuclei, to suppress non-adaptive behavior [2,3]. However, inhibitory control is not only related to brain regions but also involves other neural structures engaged in adaptive reactions to environmental stimuli, such as the Central Autonomic Network (CAN). Benarroch proposed a new model to describe the ANS-CNS (autonomic nervous system–central nervous system) two-way interaction and the continuous modulation of homeostatic processes and allostatic adaptation to internal or external requirements [4,5]. Its functional organization involves the forebrain (anterior cingulate, nucleus accumbens, insula, ventromedial prefrontal cortex, amygdala, and hypothalamus, with bidirectional interactions between rostral and caudal systems), brainstem (periaqueductal gray, parabrachial nucleus, nucleus of the solitary tract, and reticular formation of ventrolateral medulla). At the spinal level, it operates via neuronal projections of segmental reflexive ANS control. These structures receive converging visceral and nociceptive inputs (including those from thermo- and muscle receptors) and generate stimulus-specific patterns of autonomic response via projections to preganglionic sympathetic and parasympathetic neurons [4,5,6]. The forebrain and brainstem are involved in the modulation of autonomic output in response to pain and to emotional, behavioral, or “cognitive” stimuli [7,8,9].

ANS-CNS interactions emerge using different neurophysiological tools, such as repetitive Transcranial Magnetic Stimulation (rTMS) or Electroencephalography (EEG). Vernieri and colleagues [10] showed a bilateral long-lasting increase in vasomotor reactivity induced by 1-Hz rTMS accompanied by changes in the HRV that suggest a possible autonomic nervous system modulation. Low-frequency rTMS on the prefrontal cortex was also observed to induce a slight parasympathetic activation with a significant bradycardia after stimulation on the right hemisphere [11]. Finally, Triggiani and colleagues [12] found a relationship between EEG Rolandic mu rhythms recorded in a relaxed condition of resting state and HRV, suggesting a negative correlation between Rolandic low-frequency beta rhythms and sympathetic activity.

The brain controls the heart directly through the sympathetic and parasympathetic branches of the autonomic nervous system. Heart Rate Variability (HRV) describes the ANS functional setup and reflects higher brain functions, and—at least to some extent—is an independent indicator of CNS-ANS interaction [5,13,14,15]. The HRV is analyzed in time, frequency, and non-linear domains [16], and its measures reflect the activity of physiological factors modulating the heart rhythm and its adaptation to changing conditions [17,18,19]. In particular, in the non-linear domain, Sample Entropy (SampEn) [20] is a method to measure the HRV entropy quantifying the unpredictability and complexity of the interbeat intervals (IBI) series. Higher entropy indicates a more unpredictable and diverse heartbeat sequence, and lower entropy indicates a more regular and predictable heartbeat. Multiscale entropy (MSE) [21] was developed to investigate the information content in non-linear signals at different temporal scales (coarse-graining), generally using Sample Entropy. The Complexity Index (CI) is a measure of the entropy calculated from the MSE measures. It is defined as the sum of the entropies computed for different scales, providing a scalar score that allows insights into the integrated complexity of the measured system [21]. The measure of the HRV entropy has been shown to be a marker of biological systems’ health status, where higher entropy indicates better reactivity to the external/internal stimulus [22,23,24,25]. Many studies focus their attention on the HRV power frequency analysis in resting-state conditions to find the correlation between vagal activity and modulation of executive functions (see [26] for review). Furthermore, HRV was associated with the functioning of the prefrontal-subcortical circuits, with higher HRV in resting-state conditions linked to more effective prefrontal-subcortical inhibitory circuits [14,27,28]. Few works consider HRV entropy modulation during cognitive executive functions [23,29,30].

EEG is another widely used neurophysiological tool to record neural activity that can be analyzed as event-related potential (i.e., investigation of the potential fluctuation time-locked to an event) or spectral content (i.e., analysis of the neural oscillation observed in the frequency domain) [31]. In cognitive electrophysiological research, the considered frequency bands are delta (δ) (1–3 Hz), theta (ϑ) (4–7 Hz), alpha (α) (8–12), beta (β) (13–30 Hz), gamma (γ) (>30 Hz). EEG can detect general changes in the brain’s neural activity, which may not be directly attributable to specific brain regions or functions [32]. Using EEG, it is possible to observe neural abnormalities in subjects with Attention-Deficit/Hyperactivity Disorders, during eyes-open resting conditions, characterized by an increased power activity in ϑ band detected over frontal electrode sites and in the entire scalp, associated with a reduction of the α and β bands [33]. Attention and fatigue, as well as enhanced performance in attention tasks, were linked to fronto-medial activity ϑ power [34,35]. Furthermore, while α power localized in posterior regions was linked to impaired attention, it reflects improved attention when averaged across the scalp [36,37]. A complimentary role of α and ϑ oscillations in cognitive control was proposed by Gratton, where α has the role of maintaining the currently active representation association, and ϑ involved in the disruption/updating of representations when the incoming information needs attention associated [38].

Despite this evidence, no studies have investigated the intimate link and physiological activity of the Brain–Heart two-way interaction model during a motor inhibitory task. For this reason, we sought to investigate, for the first time, the correlation between spectral EEG (ϑ and α oscillations) and HRV parameters (CI and Low Frequency (LF) and High Frequency (HF) powers) during the execution of a Go/NoGo task.

We expect to find a correlation between the CI and errors, as well as a correlation between the HRV parameters and ϑ and α EEG power bands.

## 2. Materials and Methods

### 2.1. Subjects

Participants were recruited from Institute S. Anna, Crotone Italy, community recreational centers, and hospital personnel through local advertisements. Inclusion criteria were: (1) no evidence of dementia or depression symptoms according to DSM-V criteria; (2) no use of antidepressant, anxiolytic, or antipsychotic drugs that could affect cerebral blood flow; (3) right-handedness; and (4) absence of chronic medical conditions (heart disease, hypertension, or diabetes). According to these criteria, 14 right-handed healthy volunteers (8 females age 34.7 ± 11.9 and 6 males age 43.8 ± 9.2) were considered eligible for this study. All participants had normal or corrected to normal vision, and normal color vision. Before starting the recording, we ensured that the participants had not consumed caffeine or smoked in the previous four hours.

All participants gave written informed consent. The study was approved by the Ethical Committee of Regione Calabria (n.ro 172 17–July–2020.) according to the Helsinki Declaration. All subjects were advised to abstain from smoking and drinking caffeinated beverages 4–6 h before the experiment.

### 2.2. Procedure

The experiment was carried out in a sound-attenuated (below 35 dB) and dimly lit room (3 Lux) in the absence of noise or possible distractors. Participants were seated in a comfortable chair while performing a modified version of the classical Go/NoGo task, based on the Rule Shift Cards [19,39,40] optimized for EEG-ECG, which was designed using Biotrace+ (https://www.mindmedia.com, accessed on 8 May 2022, Mindmedia, version V2018A1, Roermond-Herten, The Netherlands). The Rule Shift Cards test measures cognitive flexibility and assesses the ability to shift from one rule to another in two tests presented consecutively. The protocol study was composed of a baseline lasting 5 min and two different tasks lasting 6 min each. There were 220 visual stimuli in both tasks consisting of 12 × 12 cm red, white, and chess patter squares on a black background. The first task had three different visual stimuli: red squares (frequent stimulus n.154—70%), white squares (rare stimulus n.44—20%), and chess patterns with squares (distractor n.22—10%), while in the second task, there were only red (frequent stimulus n.176—80%) and white (rare n.44—20%) squares. The interval between each stimulus was 1500 ms, with a duration of the stimulus of 500 ms.

**Figure 1:**
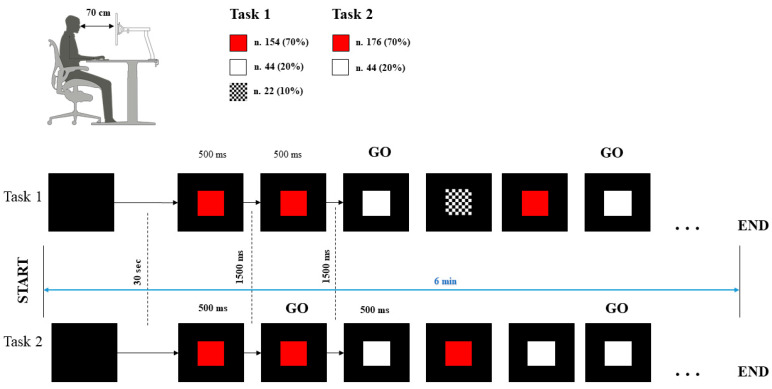
Experimental detail: The subject comfortably sits in front of the screen at 70 cm from the monitor with the hand positioned near the spacebar of the computer keyboard. In the first task, the subject must hit the spacebar when appearing the white square and stay to rest when the red square appears (the chess pattern with squares was the distractor). During the second task, the subject must hit the spacebar if the color of the square is the same as the previous square. The first square appeared after 30 s of a black image. The stimulus duration was 500 ms, and the interval of time between the stimuli was 1500 ms.

During the first task, the subject had to press the keyboard spacebar when the white square appeared, while in the second, only when two equal visual stimuli were presented consecutively (Figure 1). The sequence of stimuli was presented on a 24-inch monitor, with the subject comfortably sited at a distance of 70 cm. The setting had a constant temperature and luminosity and absence of transient noise. Before each task, the subject was instructed about the assignment, with a pause of 5 min between the two tasks.

### 2.3. Data Acquisition

The EEG and ECG recordings were made by NEXUS-32 (Mindmedia, Roermond-Herten, The Netherlands), and the stimuli were presented by Biotrace+ software (https://www.mindmedia.com/, accessed on 8 May 2022, Mindmedia, version V2018A1, Roermond-Herten, The Netherlands). Acquisitions were made at 256 Hz. A 21-channel cap with sintered Ag-AgCl ring electrodes was used for the EEG recording, together with two polygraphy channels for the ECG (recorded by adhesive electrodes positioned on the chest) and the EOG. Recording impedances were kept <5 kΩ. All signals were recorded with a common reference. Signals were filtered between 1 and 45 Hz and stored on a hard disk for offline processing. A notch filter at 50 Hz was applied.

### 2.4. Data Analysis

Five minutes of baseline and 6 min for each test of EEG signals were extracted and analyzed by EEGLAB (https://sccn.ucsd.edu/eeglab/index.php, 20 January 2021, Swartz Center for Computational Neuroscience, version 2022.0, La Jolla, CA, USA) and visually controlled for eye blink and muscular artifacts. The artifacts remotion was done using the Independent Component Analysis (ICA) method. The channels selected for the power analysis were frontal (F3, Fz, F4), central (C3, Cz, C4), parietal (P3, Pz, P4), and occipital (O1, O2). The power of α (8–12 Hz) and ϑ (4–7 Hz) EEG bands were extracted and expressed in dB (10log_10_(mV^2^/Hz)).

The ECG was analyzed by Kubios advanced software for HRV analysis (Kubios, version 3.1, Kuopio, Finland). The signals were controlled for artifacts and ectopic beats removed by the interpolation method. For the ECG analysis, the CI and natural logarithm (Ln) of HF and LF FFT spectral bands were calculated. The CI was based on the MSE approach, quantifying the degree of irregularity over a range of coarse-grained scales (τ) from 1 to 3. The coarse-grained scales were constructed by averaging the IBI/tachogram’s data points within non-overlapping windows of increasing length τ. For each coarse-grained scale, the Sample Entropy was calculated, and the CI was extracted as the sum of the Sample Entropy for each coarse-grained scale. The parameters *m* and *r* of Sample Entropy (SampEn) were set to 2 and 0.2, respectively.

### 2.5. Statistical Analysis

Because of the small sample size (N = 14), the non-parametric exact test was used for the statistical analysis [41,42,43]. This approach provides more accurate results when the sample size is small or when tables are sparse or imbalanced. The Shapiro–Wilk test was used to control the normal distribution of the data [44].

The Wilcoxon exact test compared the different phases between them for LnLF, LnHF, and CI HRV parameters and the dB values of α, ϑ, and α/ϑ ratio power band. Because of the small sample groups and the presence of outliers, the median of the total errors was used to divide the subjects into two sample groups with the same number of subjects: Good Performance (GP) below the median and Poor Performance (PP) above the median. The two groups were compared for the different tasks using the Mann–Whitney exact test. The effect size *r* was calculated as the absolute value of Z√(2N) (Wilcoxon’s test) or Z√(N) (Mann–Whitney’s test), where Z is the Z statistic of the statistical test and N is the total number of subjects. The effect size results were considered: r < 0.1 not significant; 0.1 ≤ r < 0.3 low; 0.3 ≤ r < 0.5 medium; r ≤ 0.5 high. The level of significance was set at *p* ≤ 0.05.

The Spearman correlation test analyzed the correlation between HRV and EEG parameters and the correlation of the errors with the recorded parameters.

## 3. Results

### Behavioral Data

All subjects performed the inhibitory task with sufficient performance. RT during GO/NoGo trials and % of errors during Go and NoGo trials are reported in Table 1. As expected, the RT increased in Task 2 as the numbers of errors in the Go and NoGo conditions.

**Figure 2:**
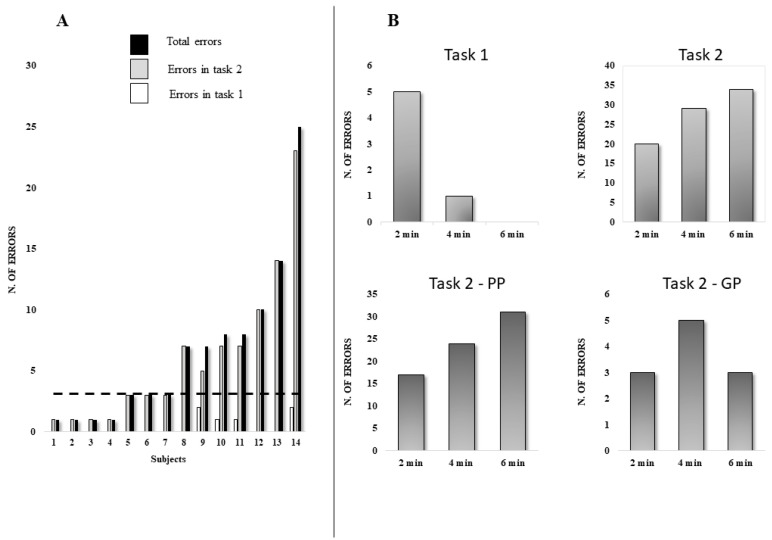
(**A**): Errors for each subject in task 1 (white), task 2 (gray), and total errors (black). The dashed line represents the median (value = 3) of the total errors. (**B**): In the first line, the histograms of the number of errors during tasks 1 and 2 for the whole group. In the second line, the histograms of the number of errors during task 2 for the PP and GP groups.

Considering the number of errors, these are lower in task 1 with a decrease over time. Conversely, in task 2, the number of errors was higher, increasing over time. However, considering task 2 and the PP and GP groups, only the first showed an increase during the task, while the performance in the GP group showed a bell trend over the task (Figure 2).

**Figure 3:**
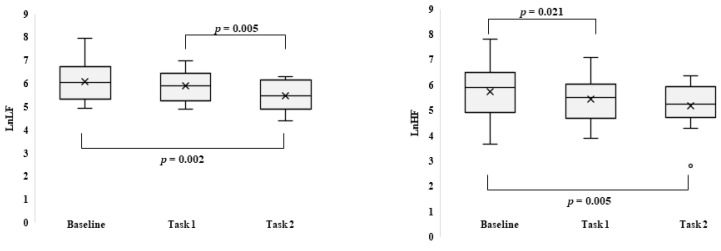
Boxplot of the natural logarithm of low-frequency power (left) and high-frequency power (right) in resting-state (baseline) and during tasks 1 and 2. In the graph, the extremities of the box represent the first (25th percentile) and the third (75th percentile) quartile, and the whiskers represent the minimum (0th percentile) and maximum (100th percentile). The central line is the median (50th percentile), the (x) is the mean, and the (o) upper or below the whiskers are the outliers.

At the physiological level, all data were normally distributed for all considered conditions (Shapiro–Wilk test: 893 ≤ W ≤ 986; 0.09 ≤ *p* ≤ 0.99). Considering the whole group, comparing the baseline with the two tasks, a significant difference was found for LnLF for LnHF only between the baseline and Task 2 (Z = −2.794, *p* = 0.002, r = 0.53 and Z = −2.48, *p* = 0.005, r = 0.47, respectively). A significant difference was also found between Task 1 and Task 2 for LnLF and LnHF (Z = −2.480, *p* = 0.005, r = 0.47; Z = −2.040, *p* = 0.021, r = 0.39) (Figure 3).

**Figure 4:**
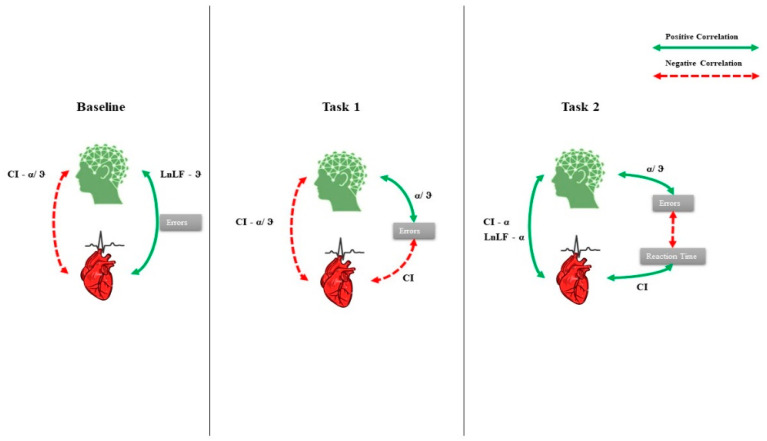
Correlation between HRV parameters (Complexity Index (CI), HF power (LnHF), LF power (LnLF)) and EEG α, ϑ, and α/ϑ ratio EEG powerbands of the whole group. Green and dashed red lines are the positive and negative correlations, respectively.

**Figure 5:**
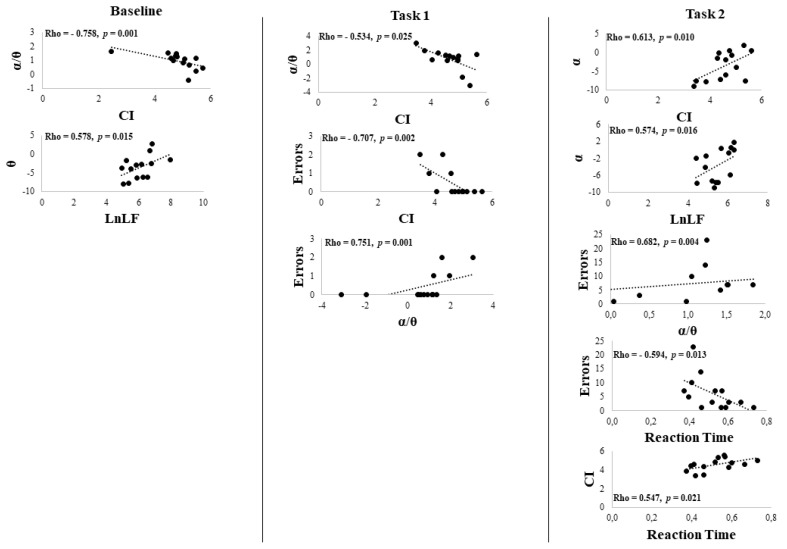
Scatter Plot of the correlation between HRV parameters (Complexity Index (CI), HF power (LnHF), LF power (LnLF)) and EEG α, ϑ, and α/ϑ ratio EEG powerbands of the whole group. The dashed line represents the tendency line.

At baseline, CI was negatively correlated with α/ϑ (Rho = −0.758, *p* = 0.001) (indicating higher α powerband) and LnLF and positively with ϑ (Rho = 0.578, *p* = 0.015). In task 1, CI was negatively correlated with the errors (Rho = −0.707, *p* = 0.002) and α/ϑ (Rho = −0.534, *p* = 0.025), while the errors were positively correlated with α/ϑ (Rho = 0.751, *p* = 0.001) (indicating lower α powerband). In task 2, CI was positively correlated with Reaction Time (Rho = 0.547, *p* = 0.021) and positively with α powerband (Rho = 0.613, *p* = 0.010). LnLF was positively correlated with α power band (Rho = 0.574, *p* = 0.016), and the errors were negatively correlated with the Reaction Time (Rho = −0.594, *p* = 0.013) and positively correlated with α/ϑ (Rho = 0.682, *p* = 0.004) (Figure 4 and Figure 5).

**Figure 6:**
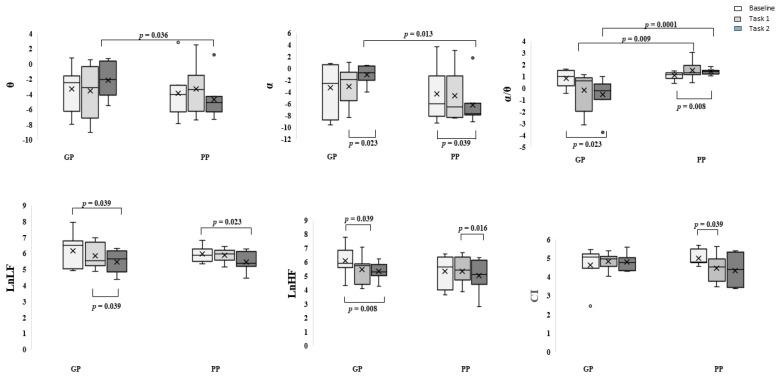
In the first line boxplot α, ϑ, and α/ ϑ ratio powerband. In the second line, the natural logarithm of Low Frequency (LF) and High Frequency (HF) and the HRV Complexity Index (CI). In light gray, the baseline, medium gray task 1, and dark gray task 2. GP the group with Good Performance; PP the group with Poor Performance. In the graph, the extremities of the box represent the first (25th percentile) and the third (75th percentile) quartile, and the whiskers represent the minimum (0th percentile) and maximum (100th percentile). The central line is the median (50th percentile), the (x) is the mean, and the (o) upper or below the whiskers are the outliers.

Comparing GP and PP groups, at Mann–Whitney exact test, a significant difference was observed for ϑ and α powerband in task 2 (Z = −1.853, *p* = 0.036, r = 0.5; Z = −2.236, *p* = 0.013, r = 0.63, respectively) with higher values for the GP group, and for α/ϑ ratio power band in tasks 1 (Z = −2.364, *p* = 0.009, r = 0.63) and 2 (Z = −3.130, *p* = 0.0001, r = 0.84) with lower values for GP group (Figure 6). No significant differences were found for HRV parameters, even if LnHF and LnLF values were higher in the GP group (Figure 6). Controlling the recorded parameters within groups, GP groups showed increasing values in α and ϑ ratio powerband from baseline to task 2, while decreasing values were for PP groups. The difference was significant at Wilcoxon exact test for α powerband for the GP group between tasks 1 and 2 (Z = −2.028, *p* = 0.023, r = 0.54) and for the PP group between baseline and task 2 (Z = −1.859, *p* = 0.039, r = 0.5). At the α/ϑ ratio power band, the GP group showed decreasing values, with a significant difference between baseline and task 2 (Z = −2.028, *p* = 0.023, r = 0.54). In contrast, PP groups showed increasing values with a significant difference between baseline and task 2 (Z = −2.366, *p* = 0.008, r = 0.63) (Figure 6). For the HRV parameters, both groups showed decreasing values. The GP group showed a significant difference between baseline and task 2 (Z = −1.859, *p* = 0.039, r = 0.5) and between task 1 and task 2 (Z = −1.859, *p* = 0.039, r = 0.5) for LnLF, and between baseline and task 1 (Z = −1.859, *p* = 0.039, r = 0.5) and task 2 (Z = −2.366, *p* = 0.008, r = 0.63) for LnHF. For the PP group, the differences were significant between baseline and task 2 (Z= −2.028, *p* = 0.023, r = 0.54) for LnLF, between task 1 and task 2 (Z= −2.197, *p* = 0.016, r = 0.59) for LnHF, and between baseline and task 1 for the CI (Z = −1.859, *p* = 0.039, r = 0.5) (Figure 6).

**Figure 7:**
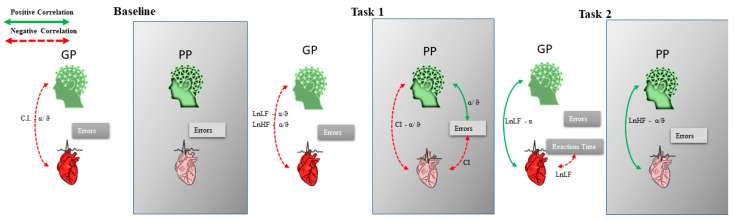
Correlation between HRV parameters (Complexity Index (CI), HF power (LnHF), LF power (LnLF)) and EEG α, ϑ, and α/ϑ ratio EEG powerbands of the group with Good Performance (GP) and of the group with Poor Performance (PP). Green and dashed red lines are the positive and negative correlations, respectively.

**Figure 8:**
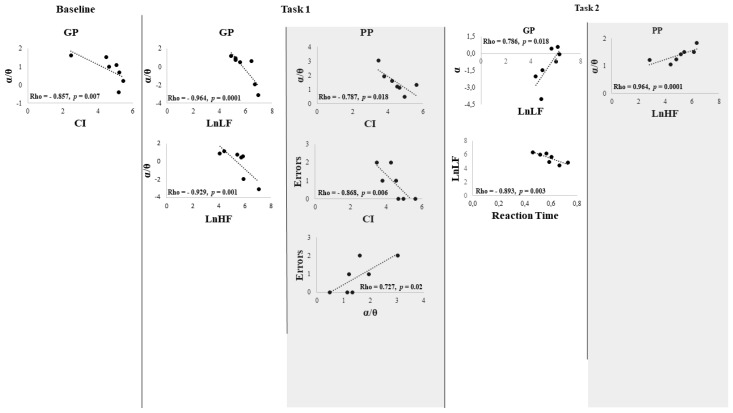
Scatter Plot of the correlation between HRV parameters (Complexity Index (CI), HF power (LnHF), LF power (LnLF)) and EEG α, ϑ, and α/ϑ ratio EEG powerbands of the group with Good Performance (GP) and of the group with Poor Performance (PP). The dashed line represents the tendency line.

The GP group showed in baseline a negative correlation between CI and α/ϑ (Rho = −0.857, *p* = 0.007). In task 1, there was a negative correlation between LnLF and LnHF with α/ϑ ratio, (Rho = −0.964, *p* = 0.0001; Rho = −0.929, *p* = 0.001, respectively), and in task 2, LnLF was negatively correlated with Reaction Time (Rho = −0.893, *p* = 0.003) and positively with power α band (Rho = 0.786, *p* = 0.018). The PP group showed no correlations at baseline. In task 1, the CI was negatively correlated with the errors (Rho = −0869, *p* = 0.006) and α/ϑ (Rho = −0.786, *p* = 0.018), while α/ϑ was positively correlated with the errors (Rho = 0.725 *p* = 0.02); in task 2, LnHF was positively correlated with α/ϑ (Rho = 0.964, *p* = 0.0001) (Figure 7 and Figure 8).

## 4. Discussion

In this study, we observed how the combined HRV and EEG parameters changed as a function of inhibitory control abilities. The electroencephalographic power bands were linked to normalized high frequency with modifications in cardiac vagal activity that showed parallel changes [45]. It has been described that high- and low-frequency components of HRV are strongly coupled with functional connectivity in the autonomic control and emotional regulation during the resting state [46,47,48]. The use of HRV entropy analysis was suggested as a biomarker of the integrated functioning of the brain [30].

To evaluate inhibitory performance, two different tasks were employed. The first task required hitting the spacebar when a white square appeared, and the second consisted of hitting the spacebar only if the colored square was equal to the previous. In the last task, the subject must modify the strategy learned in the first task to provide the correct answer, memorizing the previous appaired element and inhibiting the action in pressing the spacebar when the square appaired is different from the previous. Comparing the two tasks, a decreasing number of errors over time was observed in the first task, while an increase was observed in the second task. The augmentation of errors could be due to increased difficulty and maintaining attention on time. However, considering the groups separately, the GP group increased the errors in the first half-session of task 2 and then decreased, while the PP group in the same task showed an increase in errors.

Thayer and colleagues [28] observed that subjects with higher HRV made fewer total errors in the Go/NoGo test, interpreting the results as an increased ability to adapt the behavior to the environmental request. In our study, at baseline, the GP group showed higher values of the power spectrum of HF (LnHF = 6.1 ± 1.1) and LF (LnLF = 6.2 ± 1.1) compared to the group PP group (LnHF = 5.3 ± 1.1; LnLF = 6.0 ± 0.5), but without a significant difference and a decrease in LnHF and LnLF values from baseline to the end of the experiment. The lack of significance in the differences between the two groups could be due to the sample size. At the same time, the decrease in parasympathetic activity could be related to the variables’ reactivity during mental fatigue, as suggested by Melo and colleagues [49], as well as a higher level of anxiety could have contributed to low levels of vagal control [50,51].

It has been suggested that there is a general linkage between executive function and frontal and midbrain areas in regulating the vagal control of the heart [52,53], hypothesizing that attentional and affective regulation worked together in the process of self-regulation and goal-directed behaviors. This hypothesis was extended to attentional and cognitive processes in the absence of affective dimensions [54]. If both groups showed decreasing values in their sympathovagal components, the GP group kept a constant level in the CI, while the PP group showed a significant decrease. Furthermore, only the GP group showed in baseline a significant correlation between CI and α/ϑ ratio.

The CI, a non-linear measure of the HRV, is an index of the complex Brain–Heart two-way interaction and results reduced in pathological conditions [24,55,56]. The HRV reflects the activity of physiological factors modulating the heart rhythm. It represents an output of the Brain–Heart two-way interaction [4,57] and the organism’s capability to adapt to changing conditions [8,9,58]. In the CAN model, the forebrain and brainstem are involved in the modulation of autonomic output in response to pain and to emotional, behavioral, or “cognitive” stimuli. The sequence of heartbeats is not regular and exhibits complex fluctuations that are better described by non-linear analysis. The CI allows for measuring this complexity with the higher complexity related to a higher health status [22,25,55,59,60].

Studies found modest relationships between resting HF-HRV and performance during the Working Memory Test and the Continuous Performance Test [61]. Considering the whole group, we found that CI negatively correlated with the errors in the first task and positively correlated with the reaction time, and the reaction time negatively correlated with the errors in the second task. These results indicate a higher CI associated with lower impulsivity and fewer errors. Contextually, we also found the α/ϑ power band ratio positively correlated with the errors. It is important to note that as α and ϑ are negative values expressed in dB, then the decreasing of the α/ϑ ratio is indicative of higher α values.

Furthermore, the two groups showed different levels in α and ϑ powerband values and trends from baseline to task 2. The GP group showed higher values of α and ϑ power with an increasing trend from baseline to task 2, while the opposite was for the PP group.

Higher values of α and ϑ oscillations in the GP group suggest a more effective capability to change strategies in solving new tasks [27,62,63]. Furthermore, the higher level of theta activity could indicate the effort to maintain a good level of ‘relaxed concentration’ [64,65,66]. The opposite trend in the two groups indicates increasing attention in the GP group and decreased maintenance of attention in the PP group [67,68,69].

EEG α and ϑ oscillations have been extensively investigated concerning cognition [70,71,72,73,74]. These oscillations are the main features that dominate resting-state EEG, and their power is measured during tasks to elucidate moment-to-moment neural variability linked to specific stimuli or conditions and thought to be associated with mechanisms regulating the flow of information [75]. The posterior α has been related to the inhibition of the processing of visual stimuli, suggesting its relation to a process more oriented to limit the progression of perceptual information through the brain to avoid interfering with currently active representations [67,72,73,76]. In particular, in tasks requiring a high level of attention, posterior alpha results decrease after errors, which is presumably linked to attention adjustments [77,78,79]. In contrast, the ϑ band was associated with response to task setting with high levels of conflict or requiring updating [80,81,82,83]. Theta oscillations are largely related to the medial frontal cortex (MFC) activity, with increasing activity after error detection and reward omission [80,84]. The MFC has been found to be the center of the performance monitoring system, detecting situations in which the level of cognitive control should be increased [79,82,85] with the theta oscillation that is shown to have an adaptive role in responding to the events [86].

Gratton proposed a complementary role of α and ϑ oscillations in cognitive control, hypothesizing that EEG α has the role of maintaining the currently active representation associated, while EEG ϑ oscillations are involved in the disruption/updating of representations when the incoming information needs attention associated [38]. In this way, a dual control mechanism regulates thoughts and actions, with proactive control associated with the α oscillation, and a reactive control associated with the ϑ oscillation [87,88]. While the first reflects the sustained and anticipatory maintenance of goal-relevant information for optimal cognitive performance, protecting the processing from interference, the second (suppression of the alpha band) reflects transient stimulus-driven goal reactivation based on interference demands or episodic associations.

The positive correlation between LnLF and EEG ϑ powerband could be linked to the level of stress in the subjects [89]. However, this correlation was not found considering the GP and PP groups separately. Furthermore, the negative correlation between CI and α/ϑ ratio powerband observed in the baseline for the whole group was present only in the GP group. The inverse correlation of the CI at baseline could be interpreted as an increased level of CI correlated with an appropriate level of mental concentration and flexibility in adapting processing to changing tasks [27,62,63].

The negative correlation between CI and α/ϑ ratio was kept in task 1; furthermore, the CI was negatively correlated with the errors. In contrast, the α/ϑ ratio was positively correlated with the errors. The increased CI linked to an increased alpha level indicates a more effective behavioral response during the task [23] and more flexibility to modulate the reaction after an error [90]. The same correlations were found for the PP group in the task 1. Conversely, the GP group showed a negative correlation of the α/ϑ ratio with LnLF and LnHF, evidencing higher values of α powerband and better inhibitory control [77,91], linked to higher sympathovagal values [27].

During task 2, in the whole group, the CI and LnLF were positively correlated to the α power band; more CI was positively correlated with the reaction time, and the reaction time negatively within the errors, while the α/ϑ ratio, as in task 1, was positively correlated with the errors. The results suggest that the positive correlation between inhibitory control and higher brain–heart two-way interaction (expression of higher integration in the functional setup of the CAN) is indicative of better performance in the tasks. The positive correlation between LnLF and α power band could be explained as an adaptive state associated with a demand for higher energetic resources due to the increased difficulty of the test [27].

Considering the two groups separately, while the correlation between LnLF and α powerband was present in the GP group, the direct correlation between LnHF and the α/ϑ ratio in the PP group showed an increase in ϑ power linked to the parasympathetic system. A more negative correlation was found between LnLF and Reaction Time for the GP group. These results contrast with the assumption of a positive correlation between higher levels of cardiac vagal tone and cognitive performance [92,93,94]. However, positive correlations between higher levels of cardiac vagal tone and the number of errors were described in resting-state and task period [95,96], evidencing that the association between resting HRV and executive function can depend on the requirements of the cognitive test [27,95].

### Limitations

Some limitations should be considered regarding the study. Firstly, the study sample size was relatively small, although rigorously analyzed according to established standardized procedure, which ensures consistency in data collection. Second, the lack of a neuropsychological assessment at the baseline would have been useful to evaluate the relationship between performance during the Go/NoGo task with psychological traits (such as Impulsivity).

## 5. Conclusions

In this study, for the first time in our knowledge, a correlation was described between the CI of cardiac activity and the EEG α/ϑ ratio powerband during the resting state and Go/NoGo tasks.

The observed positive correlation indicates that a higher functional setup of CAN is linked to a more effective inhibitory control and better performance. Only in the GP group was the correlation between CI and α/ϑ ratio observed in the resting state, and contextually, this group showed increasing value from the baseline to the final task in α and ϑ powerband. These findings evidenced a correlation between the CAN functional setup and higher capability to sustain the stress by adapting an effective strategy of inhibitory control during the tasks. These results are consistent with the complementary role of α and ϑ oscillations in cognitive control [27,88]. Better generalization of the results by a bigger subject sample could provide new information and new insight on the brain–heart two-way interaction in the inhibitory control.

## Figures and Tables

**Table 1:** Behavioral Variables.

	Task 1	Task 2
	Mean ± SD	Mean ± SD
RT during GO trials (sec)	0.425 ± 0.11	0.52 ± 0.11
RT during NoGO trials (sec)	0.27 ± 0.05	0.42 ± 0.059
% errors during GO trials	0.09%	2.01%
% errors during NoGo trials	0.38%	3.23%

## Data Availability

The data presented in this study are available on request from the corresponding author.
